# Transient Ischemic Attack Shakes: A Case Report

**DOI:** 10.7759/cureus.28410

**Published:** 2022-08-25

**Authors:** Sheri P Walls, Tinuola Andre, Alexander Adetunji, Eunice Hama

**Affiliations:** 1 Internal Medicine, Piedmont Athens Regional, Athens, USA; 2 Internal Medicine, Ross University School of Medicine, Athens, USA

**Keywords:** bilateral carotid stenosis, tia shake, and transient ischemic attack (tia), tia, stroke

## Abstract

Transient ischemic attack (TIA) shakes can present as epilepsy which could lead to misdiagnosis. When a patient present with neurological findings we must ensure our differentials remain broad. As a physician, we must ensure our role in analyzing the full clinical picture of our patients. We present the case of a 75-year-old man with multiple comorbidities who presented with limb shaking and seizure-like symptoms and who was found to have bilateral carotid stenosis. After finding bilateral carotid stenosis, it ultimately led to the diagnosis of “TIA Shakes.” Overall, this case re-emphasizes the importance of diagnosis and correctly managing our patients.

## Introduction

Limb shaking transient ischemic attacks (TIAs) are a rare manifestation of carotid-occlusive disease. The symptoms include a seizure-like activity which is often misdiagnosed as focal motor seizures [1,2]. A careful history may provide important clinical clues to help differentiate it from focal seizures including the absence of a Jacksonian march or aura and precipitation by maneuvers that lead to carotid compression [1,2]. TIA is a cause of the major risk of stroke. TIA is reported in the United States to be around 2.3% [3,4]. Common risk factors for TIA's are multifactorial they are not limited but include metabolic syndrome, alcohol use disorder, obesity, myocardial infarction, hypercoagulable states, and left atrial enlargement [4]. Pathogenesis of TIA has several mechanisms that range from large-artery atherosclerosis, cardio-embolism systemic embolization, and small vessel occlusion [5,6]. We present the case of an elderly gentleman with recurrent limb shaking TIAs initially diagnosed as epilepsy. His symptoms did not respond to optimization of his blood pressure but did respond to lifestyle modifications and avoidance of precipitants. The case report highlights the importance of accurate diagnosis as treatment of the associated carotid artery occlusion may help reduce the risk of future strokes.

## Case presentation

A 75-year-old male with a history of basal cell carcinoma, end-stage renal disease on nightly peritoneal dialysis, and hypertension presented with a two-month history of falls and repetitive less than 60-second limb jerking movements involving both upper and lower extremities bilaterally that resulted in the loss of posture leading to multiple falls. There was no preceding aura, loss of consciousness, fecal or urinary incontinence, or tongue biting. About two months prior to the presentation, he described an increased frequency of falls that were not related to the position. However, 72 hours prior to presentation the falls increased in frequency associated with 20-30-second episodes of alternating unilateral and or bilateral lower and upper extremity jerks and weakness. Throughout these episodes, the patient did not lose consciousness, did not have urinary or fecal incontinence, nor described any other symptoms. Due to the progression in his symptoms, he was evaluated by his primary care team, neurology, and cardiology but the workup including Holter monitor, MRI of the brain, and continuous EEG was unrevealing.

**Figure 1:**
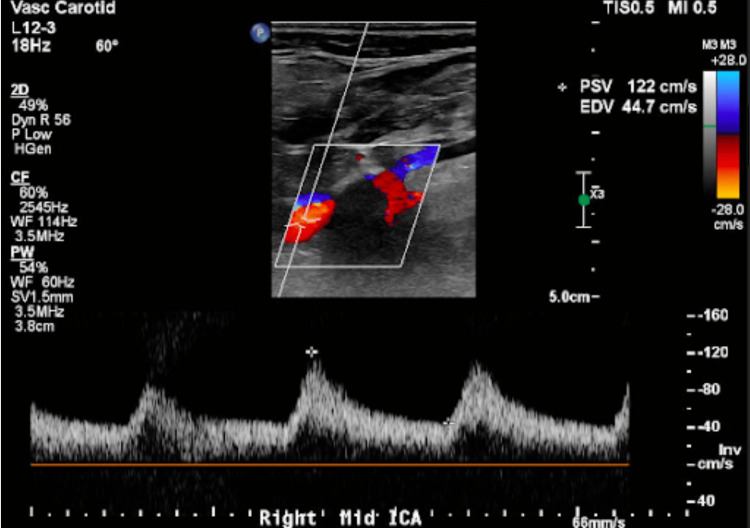
Right proximal internal carotid artery and bifurcation with severe >70% stenosis

**Figure 2:**
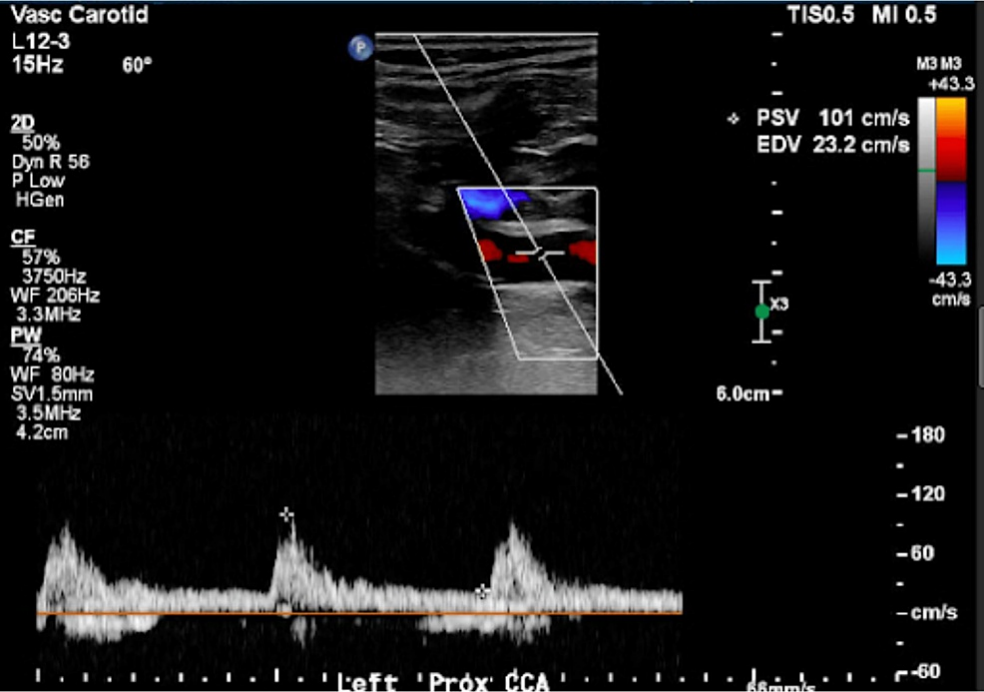
Left internal carotid artery revealed significant (50-69%) stenosis

While in the hospital, he was further evaluated with a carotid ultrasonogram which revealed right proximal internal carotid artery/bifurcation with severe (>70%) stenosis (Figure 1). The left internal carotid artery also revealed significant (50%-69%) stenosis (Figure 2).

**Figure 3:**
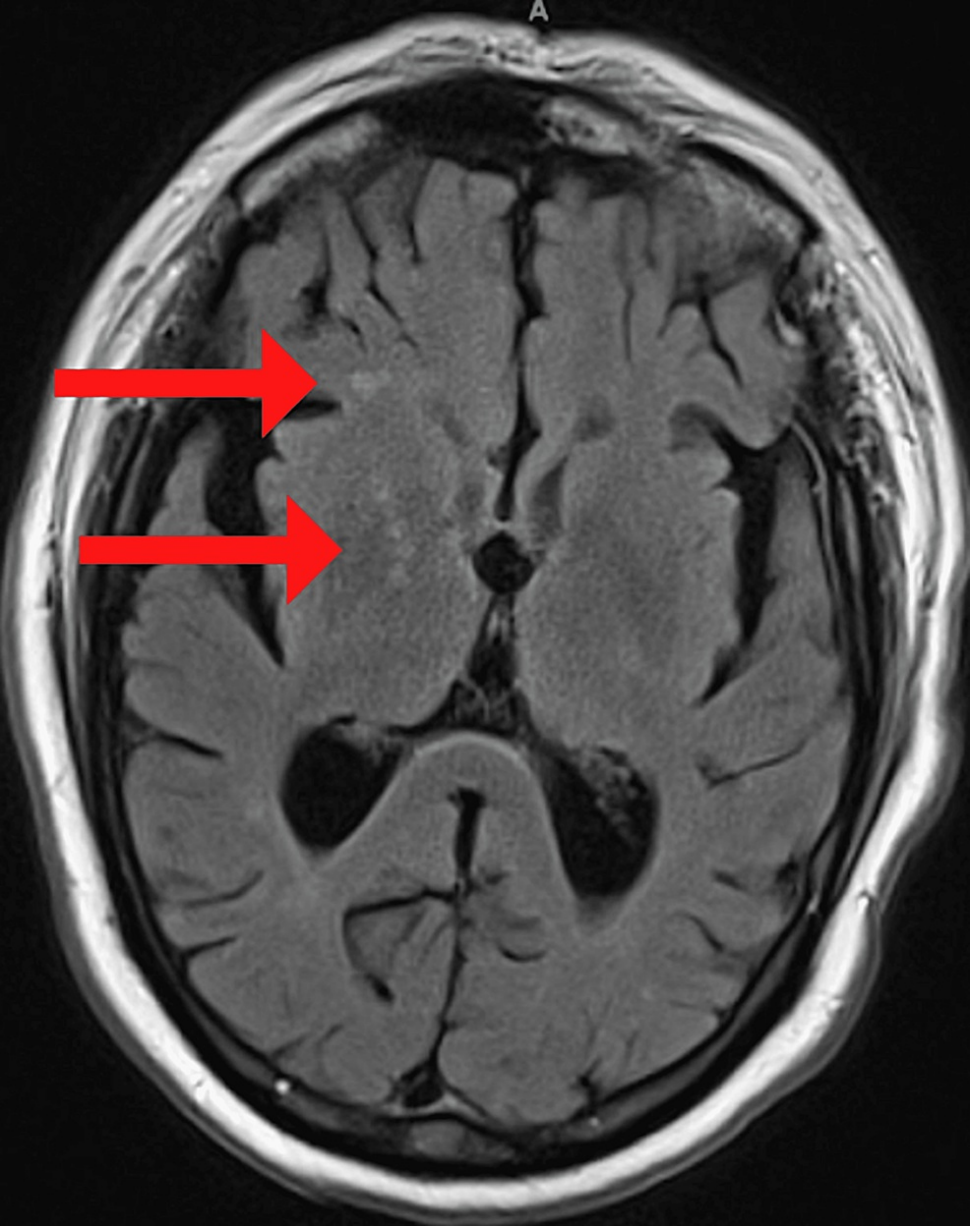
Right MCA distribution infarct MCA - Middle Cerebral Artery

He subsequently developed episodic weakness of the left upper and lower extremities. MRI of the brain without contrast found a right middle cerebral artery distribution infarct (Figure 3). He was reviewed by vascular surgery with recommendations for carotid revascularization after cardiac clearance. He was reviewed by vascular surgery who recommended the patient follow up as an outpatient after cardiac risk assessment for carotid revascularization and computed tomography of the head and neck. He was also advised to continue aspirin 81 mg and atorvastatin 40 mg therapy. He underwent carotid endarterectomy after cardiac clearance with intraoperative right carotid duplex and shows no evidence of B-mode or doppler defects involving the internal carotid artery or external carotid artery. There was a residual plaque in the proximal right common carotid artery end point creating an approximately 50% stenosis. Postoperatively, he developed a right neck hematoma which was managed conservatively and was discharged home to follow up as an outpatient.

## Discussion

Cardiovascular strokes are one of the leading causes of death, disability, and depression in the United States [1]. Cardiovascular strokes are defined as acute onset focal neurological deficit caused by several etiologies, hemorrhagic, ischemic, embolic, and cryptogenic. TIAs were previously defined as focal neurological deficits that last less than 24 hours, however, that definition has recently changed [1,2]. TIA is distinguished from stroke by the absence of abnormalities on neuroimaging, including noncontract CT of the head and/or MRI.

In 1962, Miller Fisher first described limb shaking TIA as transient rhythmic jerking [3-6]. The TIA shakes can be unilateral, bilateral, or contralateral and may be associated with rhythmic jerks that are very similar to seizures. Certain features and testing may help distinguish seizures from TIA shakes. In TIA shakes symptoms may be worsened by position such as hyperextension of the neck, walking, and standing. With these episodes which usually last than 5 minutes, the patient typically remains aware, fully oriented, and conscious and the symptoms may be relieved by either being supine or sitting down. However, with focal seizures patients may exhibit auras, they can be precipitated by hyperventilation, bilateral arm flexion and extension, brief staring episodes, and followed by a post-ictal state for minutes to hours. With respect to electrocardiography testing, the results are negative and show no abnormalities typical of seizure disorders.

## Conclusions

In summary, TIA shakes are a relatively rare manifestation of carotid disease. The importance of being able to recognize this condition is that it is often misdiagnosed as focal motor seizures. It is always important to take a thorough history and exam to have the full clinical picture to accurately diagnose and treat patients in order to decrease the chances of a major ischemic event. 
